# TEA–NaOAC Synergistic System for Hydrothermal Synthesis of Fe_3_O_4_ with Tunable Morphologies from a Single Iron Source

**DOI:** 10.3390/molecules31091463

**Published:** 2026-04-28

**Authors:** Chang Chen, Yaohui Xu, Qin Wang, Zhao Ding

**Affiliations:** 1 Laboratory for Functional Materials, School of New Energy Materials and Chemistry, Leshan Normal University, Leshan 614000, China; chenchang@lsnu.edu.cn (C.C.); wq306115@lsnu.edu.cn (Q.W.); 2 Leshan West Silicon Materials Photovoltaic and New Energy Industry Technology Research Institute, Leshan 614000, China; 3 National Engineering Research Center for Magnesium Alloys, College of Materials Science and Engineering, Chongqing University, Chongqing 400044, China

**Keywords:** Fe_3_O_4_, hydrothermal synthesis, triethanolamine, sodium acetate, morphology control, magnetic properties

## Abstract

Achieving tunable morphologies of Fe_3_O_4_ using a single iron source remains challenging, mainly due to the oxidation of Fe^2+^ and the difficulty of regulating anisotropic crystal growth. In this study, Fe_3_O_4_ was synthesized via a one-step hydrothermal method using FeSO_4_·7H_2_O as a single iron source in a TEA–NaOAC synergistic system. The effects of hydrothermal temperature, additive ratio, and dosage were systematically investigated. Time-dependent and TEA dosage-dependent experiments were also designed to elucidate the morphological evolution mechanism. The results show that pure-phase Fe_3_O_4_ can be obtained with TEA alone, as TEA controls the release rate of Fe^2+^ and inhibits its oxidation. However, the synergistic addition of NaOAC provides a mild alkaline environment that not only maintains phase purity but also further promotes anisotropic crystal growth and enables a broader morphological tunability. By tuning the reaction conditions, a systematic morphological evolution from flower-like to cubic, regular octahedral, and polyhedral structures was achieved. Time-dependent experiments reveal a complete dissolution–recrystallization pathway from flower-like to cubic structures. TEM and SAED confirm that the polyhedral particles are micrometer-sized single crystals. Under optimized conditions (160 °C, TEA 3 mL, NaOAC 26 mmol), the polyhedral Fe_3_O_4_ exhibits a saturation magnetization of 91.4 emu/g, approaching the bulk theoretical value (92 emu/g), and a coercivity of approximately 100 Oe. This study provides new experimental evidence for morphology regulation of Fe_3_O_4_ using a single iron source, achieving high saturation magnetization close to the bulk theoretical value and moderate coercivity suitable for non-biomedical applications.

## 1. Introduction

Fe_3_O_4_, an important magnetic material with an inverse spinel structure (space group Fd–3m), has shown broad application prospects in fields such as catalysis [1], magnetic separation [2], drug delivery [3], magnetic resonance imaging [4], and lithium–ion batteries [5] due to its excellent magnetic properties, good biocompatibility, low toxicity, and chemical stability [6]. Studies have demonstrated that the morphology, size, and crystallinity of Fe_3_O_4_ have a decisive influence on its properties [7]. For instance, one-dimensional nanostructures facilitate electron transport, two-dimensional sheet-like structures benefit surface reactions, and three-dimensional polyhedral structures may exhibit enhanced magnetic anisotropy due to the exposure of specific crystal facets [8,9]. Therefore, achieving morphology regulation of Fe_3_O_4_ morphology is not only an important topic in the field of materials synthesis but also crucial for expanding its application scope.

The hydrothermal method has been widely used for the synthesis of Fe_3_O_4_ due to its simple operation, and the high crystallinity of the products [10,11]. Traditional hydrothermal methods typically employ mixed Fe^3+^ and Fe^2+^ salts as iron sources, obtaining pure-phase Fe_3_O_4_ by adjusting their molar ratio [12]. However, this approach suffers from drawbacks such as a complex system and the susceptibility of Fe^2+^ to oxidation. In recent years, research using single Fe^2+^ salts as iron sources has gradually attracted attention because it simplifies the reaction system [13]. Nevertheless, the use of a single Fe^2+^ salt faces two major challenges: first, Fe^2+^ is highly prone to oxidation under hydrothermal conditions, generating impurity phases such as Fe_2_O_3_ or FeOOH [14]; second, in the absence of Fe^3+^ participation, regulating the nucleation and growth of crystals to obtain desired morphologies Fe_3_O_4_ imposes higher demands on the design of additives [15].

To address these issues, the selection of appropriate additives is crucial [16,17]. Triethanolamine (TEA) is a commonly used complexing agent, in which the nitrogen atom and hydroxyl oxygen atoms can form stable five-membered ring complexes [Fe(TEA)]^2+^ with Fe^2+^, thereby effectively controlling the release rate of Fe^2+^ and inhibiting its oxidation. Simultaneously, the slow dissociation characteristic of TEA facilitates “rate-controlled synthesis”, creating conditions for orderly crystal growth [18]. Anhydrous sodium acetate (NaOAC) serves as a mild alkali source, hydrolyzing under hydrothermal conditions to provide OH^−^ and creating a suitable alkaline environment to promote the nucleation of Fe_3_O_4_ [19]. Furthermore, Ac^−^ ions are considered capable of selectively adsorbing onto specific crystal facets of Fe_3_O_4_, thereby influencing its anisotropic growth and playing an auxiliary role in morphology regulation [20]. Therefore, the combination of TEA and NaOAC holds promise for achieving dual regulation of “rate control” and “orientation control” in a single iron source system, obtaining pure-phase Fe_3_O_4_ with tunable morphology. Although a few studies have involved the application of TEA or NaOAC in the synthesis of Fe_3_O_4_, most have been limited to preliminary explorations of single additives, and systematic investigations into their synergistic effects remain scarce. In particular, how factors such as temperature, TEA dosage, and NaOAC dosage collectively influence the phase composition, crystallinity, microstrain, and morphological evolution of Fe_3_O_4_, as well as the underlying “dissolution–recrystallization” mechanism, have yet to be thoroughly elucidated.

Although the TEA-NaOAC system has been reported for the synthesis of Fe_3_O_4_, previous studies have primarily focused on morphological characterization and magnetic property measurement under fixed conditions. Systematic investigations of key parameters such as temperature, time, and additive dosage, as well as the kinetics of morphological evolution, have been less explored. Specifically, Chen et al. [18] obtained Fe_3_O_4_ particles with different morphologies by varying the TEA/NaOAC ratio, whereas the independent effect of hydrothermal temperature (120–200 °C) and the time-dependent morphological evolution in the TEA-only system were not the main focus of that study. In addition, fine structural characterizations such as TEM and XPS were not included in that work, leaving the single-crystal or polycrystalline nature of the particles unaddressed, and the distinction between Fe_3_O_4_ and *γ*-Fe_2_O_3_ was not discussed. Compared with the work by Wang et al. [21], which achieved high Ms Fe_3_O_4_ without TEA/NaOAC, the present work focuses on morphology regulation while maintaining good magnetic performance.

In the present work, we aim to complement and extend previous studies by providing a more systematic investigation. We examine the synergistic and individual roles of TEA and NaOAC over a wide temperature range (120–200 °C), clarifying the distinct functions of the two additives through control experiments (TEA alone, NaOAC alone, and additive-free). We design time-dependent (1–72 h) and TEA dosage-dependent (1–5 mL) experiments in the TEA-only system to reveal the dissolution–recrystallization pathway from flower-like to cubic structures, and to identify the TEA dosage range favorable for regular octahedra at 200 °C. Using HRTEM, SAED, XPS and FTIR, we characterize the polyhedral Fe_3_O_4_ obtained in the TEA-NaOAC system and confirm its single-crystal nature as well as the effective removal of organic residues. Finally, we compare the magnetic properties of four typical morphologies (flower-like, cubic, regular octahedral, and polyhedral) to establish a morphology–magnetic property relationship. By adjusting the hydrothermal temperature, reaction time, or TEA/NaOAC dosage, the morphology can be systematically tuned among these four distinct types. This work is expected to provide a more comprehensive experimental basis for the controllable preparation of Fe_3_O_4_ using a single iron source.

## 2. Results and Discussion

### 2.1. Phase Analysis and Morphological Observation (TEA + NaOAC, Temperature Gradient)

**Figure 1:**
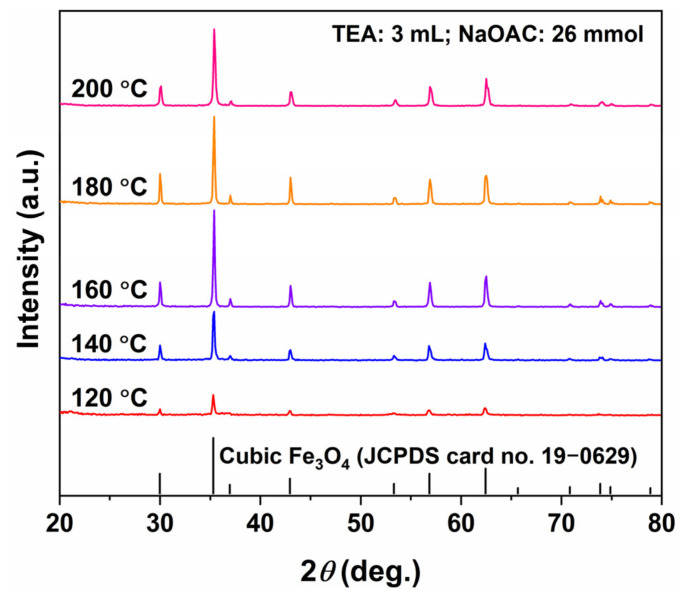
XRD patterns of Fe_3_O_4_ samples synthesized at different hydrothermal temperatures for 12 h in the presence of both TEA (3 mL) and NaOAC (26 mmol).

Figure 1 shows the XRD patterns of the products synthesized hydrothermally at different temperatures (120–200 °C) for 12 h in the presence of both TEA (3 mL) and NaOAC (26 mmol). All diffraction peaks can be indexed to Fe_3_O_4_ with an inverse spinel structure (JCPDS No. 19-0629), and no additional peaks corresponding to other phases such as FeOOH or Fe_2_O_3_ are detected, indicating that pure Fe_3_O_4_ is obtained under all experimental conditions. Notably, the diffraction peak intensity is weakest for the Fe_3_O_4_ obtained at 120 °C. As the temperature increases to 140 °C and 160 °C, the peak intensity increases significantly, reflecting an improvement in crystallinity. However, when the temperature further rises to 180 and 200 °C, the increase in peak intensity becomes less pronounced, suggesting that the crystal growth and crystallization optimization of Fe_3_O_4_ are essentially completed within this temperature range.

**Figure 2:**
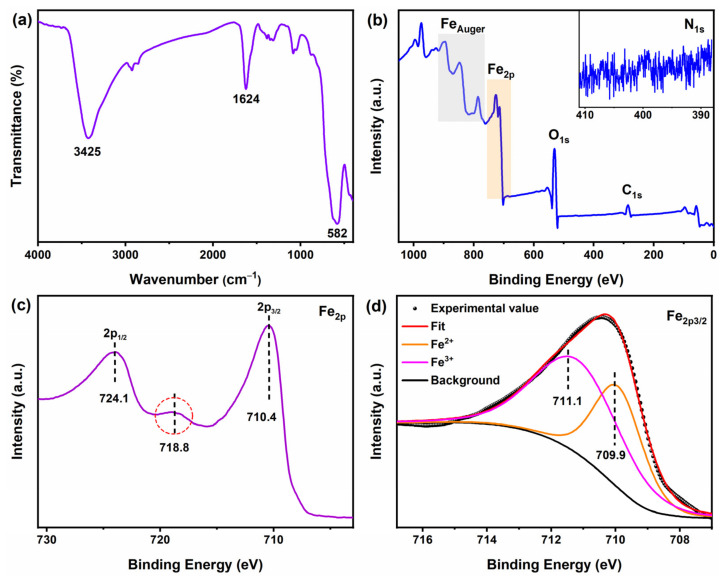
Characterization of the Fe_3_O_4_ sample synthesized at 160 °C for 12 h with TEA (3 mL) and NaOAC (26 mmol): (**a**) FTIR spectrum, (**b**) XPS survey spectrum (inset: high-resolution N 1s spectrum), (**c**) high-resolution Fe_2p_ spectrum, and (**d**) multiplet fitting of the Fe_2p3/2_ region decomposed into two main components (Fe^2+^ and Fe^3+^).

Figure 2 presents the characterization results of the Fe_3_O_4_ sample synthesized at 160 °C for 12 h with TEA (3 mL) and NaOAC (26 mmol). As shown in the FTIR spectrum in Figure 2a, the absorption bands at 3425 cm^−1^ and 1624 cm^−1^ are assigned to O–H stretching vibrations of surface hydroxyl groups and bending vibrations of adsorbed water, respectively [19]. A strong and broad band at approximately 582 cm^−1^ is observed, which is characteristic of the Fe–O stretching vibration in Fe_3_O_4_ (tetrahedral Fe^3+^–O) [22]. Notably, no characteristic multi-peaks of *γ*-Fe_2_O_3_ (at ~640, 660, 700 cm^−1^) or the doublet peaks of *α*-Fe_2_O_3_ (at ~470, 540 cm^−1^) are detected, strongly supporting the formation of pure-phase Fe_3_O_4_ [23]. Moreover, no obvious absorption peaks are observed at around 2950 cm^−1^ (C–H stretching) or 1560 cm^−1^ (asymmetric –COO^−^ stretching), indicating that organic residues from TEA and NaOAC have been effectively removed by repeated washing with ethanol and water. Figure 2b displays the XPS survey spectrum, which shows characteristic peaks of Fe_Auger_, Fe_2p_, O_1s_, and C_1s_. The inset in Figure 2b is the high-resolution N 1s spectrum, which is dominated by background noise with no detectable N 1s peak, further confirming the very low residual of TEA after washing.

Figure 2c shows the high-resolution Fe_2p_ spectrum, which exhibits typical spin–orbit doublets: Fe_2p3/2_ at approximately 710.4 eV and Fe_2p1/2_ at approximately 724.1 eV, with a splitting energy of about 13.7 eV. A distinct broad peak on the higher binding energy side of the Fe_2p3/2_ main peak (at about 718.8 eV) is assigned to the shake-up satellite of Fe^2+^. This satellite feature is a key fingerprint distinguishing Fe_3_O_4_ from *γ*-Fe_2_O_3_, since *γ*-Fe_2_O_3_ contains only Fe^3+^ and does not exhibit such a satellite structure [22]. A similar spectral feature, particularly the pronounced shoulder between the two main peaks, has been observed in Fe_3_O_4_-based nanocomposites reported in the literature [24], further supporting the assignment of the satellite to Fe^2+^ species. Figure 2d presents the multiplet fitting results of the Fe_2p3/2_ region. Following the method proposed by Biesinger et al. [25], the Fe_2p3/2_ peak is decomposed into two main components: a peak at approximately 709.9 eV with a narrower full width at half maximum (FWHM) assigned to octahedral Fe^2+^, and a peak at approximately 711.1 eV with a broader FWHM assigned to tetrahedral/octahedral Fe^3+^. Similar fitting approaches have been reported for Fe_3_O_4_ nanoparticles [26]. The fitting results clearly confirm the coexistence of Fe^2+^ and Fe^3+^ in the sample. Combined with the FTIR results and the clear presence of the Fe^2+^ satellite peak, the main phase of the product is confirmed to be Fe_3_O_4_.

**Figure 3:**
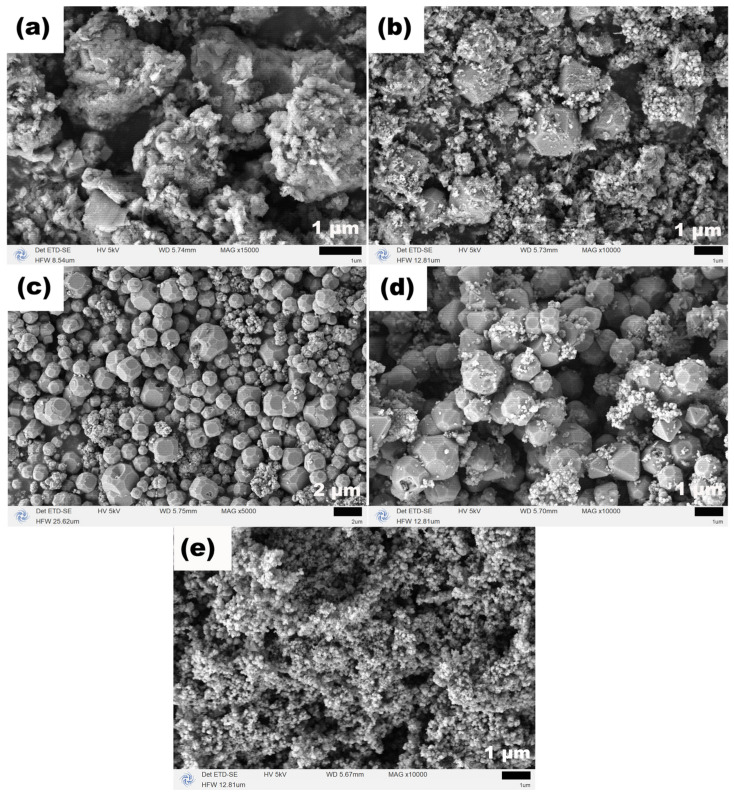
SEM images of Fe_3_O_4_ samples synthesized at (**a**) 120, (**b**) 140, (**c**) 160, (**d**) 180 and (**e**) 200 °C for 12 h in the presence of both TEA (3 mL) and NaOAC (26 mmol).

Figure 3 presents the SEM images that vividly illustrate the temperature-driven morphological evolution, providing direct evidence for the “dissolution–recrystallization” mechanism discussed above. At 120 °C (Figure 3a), the Fe_3_O_4_ mainly consists of irregular aggregates approximately 2 μm in size. Close observation reveals that these aggregates are assembled from numerous nanosized subunits with nascent polyhedral features. As the temperature rises to 140 °C (Figure 3b), the morphology becomes more complex, with the coexistence of well-defined polyhedra around 2 μm in size and aggregates composed of nanoscale polyhedral particles, indicating ongoing crystal growth and self-assembly processes. At 160 °C (Figure 3c), the Fe_3_O_4_ is predominantly composed of relatively uniform octahedral particles with sizes ranging from 0.5 to 4 μm, although a small number of residual aggregates formed by nano-polyhedra can still be observed. This morphology suggests that crystal growth and oriented attachment have reached a relatively balanced state. However, when the temperature reaches 180 °C (Figure 3d), numerous dispersed nanoparticles begin to appear around the micron-sized polyhedra, signaling a new dynamic change. The most significant transformation occurs at 200 °C (Figure 3e), where the previously dominant micron-sized polyhedra almost completely disappear, replaced by a large number of irregular nanoparticles (approximately 150 nm) and their aggregates. This dramatic transition strongly confirms that at excessively high temperatures, the directing effect of TEA and NaOAC on crystal growth may be weakened, leading to partial dissolution of the pre-formed micron-sized polyhedra and driving the system toward a new kinetic equilibrium dominated by nanoscale particles and their aggregates under such extreme conditions. Collectively, this morphological evolution sequence delineates a complete dynamic pathway from “nucleation and initial assembly” through “oriented growth and self-assembly” to “high-temperature-induced dissolution and reconstruction”.

**Figure 4:**
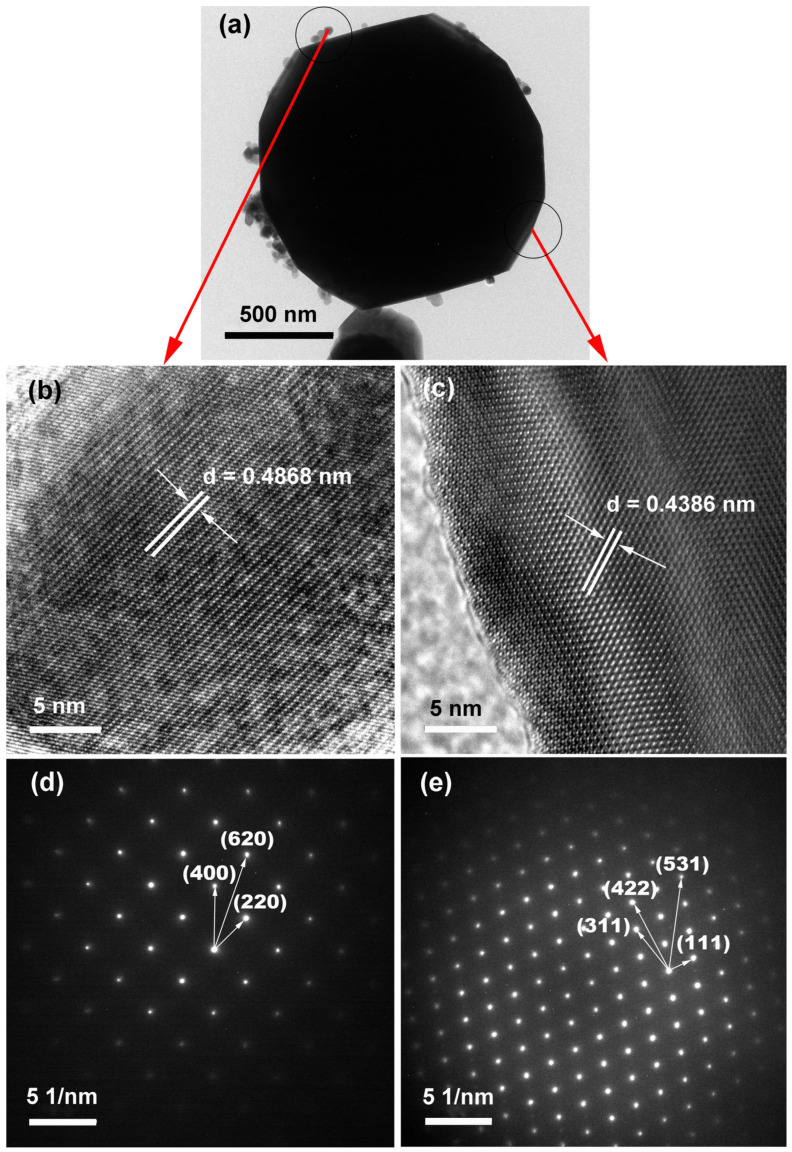
TEM analysis of the Fe_3_O_4_ sample synthesized at 160 °C for 12 h with TEA (3 mL) and NaOAC (26 mmol): (**a**) low magnification TEM image; HRTEM images of the (**b**) fragmented nanoparticle region and (**c**) polyhedral particle region; SAED patterns of the (**d**) fragmented nanoparticle and (**e**) polyhedral particle.

The Fe_3_O_4_ sample synthesized at 160 °C with TEA (3 mL) and NaOAC (26 mmol) was further characterized by TEM, and the results are presented in Figure 4. The low magnification image (Figure 4a) shows a complete polyhedral particle with a few fragmented nanoparticles attached on its surface and surroundings. HRTEM images (Figure 4b,c) reveal clear and continuous lattice fringes for both types of particles; notably, the polyhedral particle exhibits atomic scale lattice fringes where individual atomic columns can be resolved, indicating that both are high quality single crystals. The measured lattice spacing of the fragmented nanoparticles (0.4868 nm) agrees well with the d value of the (111) planes of Fe_3_O_4_ (0.4852 nm, JCPDS No. 19-0629), while that of the polyhedral particle (0.4386 nm) is slightly smaller. This deviation is most likely attributable to residual compressive strain within the polyhedral particle, which induces lattice contraction along specific directions. The SAED patterns (Figure 4d,e) further confirm the single-crystalline nature. The pattern of the fragmented nanoparticle (Figure 4d) exhibits sharp discrete spots that can be indexed to the (220), (400), and (620) planes of inverse spinel Fe_3_O_4_ (JCPDS No. 19-0629). In contrast, the pattern of the polyhedral particle (Figure 4e) shows spots indexed to the (111), (311), (422), and (531) planes. These two distinct sets of diffraction spots indicate that the two types of particles have different crystallographic orientations. Nevertheless, both patterns display sharp discrete spots, ruling out the possibility of polycrystalline aggregates, and the sharper spots of the polyhedral particle reflect its higher crystalline perfection. Despite this difference in orientation and lattice spacing, both HRTEM and SAED data unequivocally confirm that both the fragmented nanoparticles and the polyhedral particles are single crystalline Fe_3_O_4_, and no other iron oxide phases are detected in the analyzed regions.

### 2.2. Synergistic and Individual Roles of TEA and NaOAC

#### 2.2.1. Effect of NaOAC Alone (Without TEA)

To elucidate the respective roles of TEA and NaOAC in the formation of Fe_3_O_4_, comparative experiments were conducted in the absence of either TEA or NaOAC, systematically investigating their effects on the phase composition and morphology of the products.

**Figure 5:**
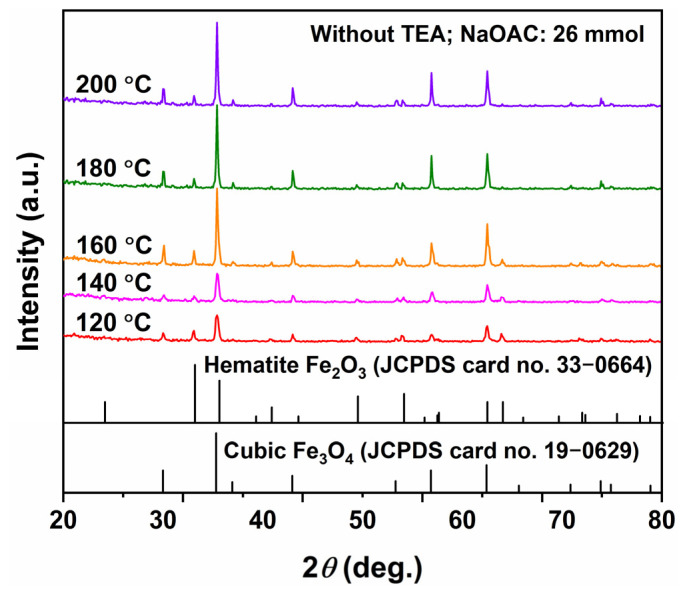
XRD patterns of samples synthesized at different hydrothermal temperatures for 12 h with NaOAC (26 mmol) and without TEA.

Figure 5 shows the XRD patterns of the samples synthesized at different temperatures with the addition of NaOAC (26 mmol) but without TEA. The main diffraction peaks of all samples could be indexed to the inverse spinel Fe_3_O_4_ phase (JCPDS No. 19-0629). The products obtained at 120 °C and 140 °C exhibited similar diffraction patterns with relatively low peak intensities. When the temperature increased to 160 °C, the diffraction peak intensity of Fe_3_O_4_ increased significantly, while further elevation of the temperature beyond 160 °C resulted in a gradual plateau in peak intensity. Notably, additional diffraction peaks were clearly observed in all XRD patterns, which were consistent with the standard pattern of hematite (*α*-Fe_2_O_3_, JCPDS No. 33-0664). This indicates that, in the absence of TEA, the products obtained at all hydrothermal temperatures consisted of a mixture of Fe_3_O_4_ and *α*-Fe_2_O_3_. These results demonstrate that TEA plays a crucial role in maintaining the formation of pure-phase Fe_3_O_4_; its absence leads to the oxidation of some Fe^2+^ ions during the hydrothermal process, resulting in the generation of Fe_2_O_3_ as an impurity phase.

**Figure 6:**
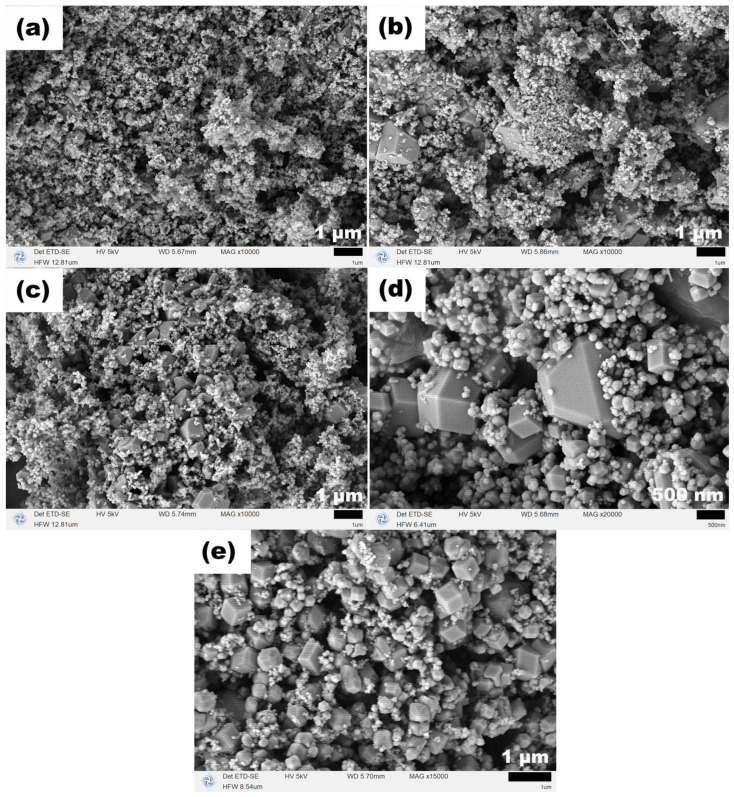
SEM images of samples synthesized at (**a**) 120, (**b**) 140, (**c**) 160, (**d**) 180 and (**e**) 200 °C for 12 h with NaOAC (26 mmol) and without TEA.

Figure 6 displays the SEM images of the corresponding samples, illustrating the morphological evolution with temperature. At 120 °C, the product mainly consisted of nanoparticles approximately 120 nm in diameter, interspersed with a small number of polyhedral particles around 1 μm in size. As the temperature increased to 140 and 160 °C, the proportions of polyhedral particles and nanoparticles became comparable, resulting in a mixed morphology. When the temperature was further raised to 180 and 200 °C, the polyhedral morphology gradually dominated, with all particle sizes remaining below 1 μm. This morphological evolution indicates that, in the presence of NaOAC alone and without TEA, elevated temperatures favor the formation of polyhedral particles. However, the products consistently remained as two-phase mixtures, and pure-phase Fe_3_O_4_ could not be obtained under these conditions.

#### 2.2.2. Effect of TEA Alone (Without NaOAC)

**Figure 7:**
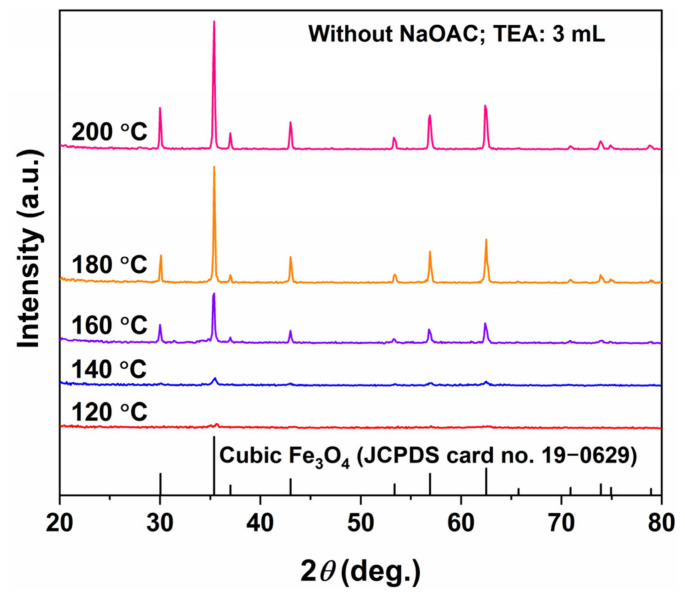
XRD patterns of samples synthesized at different hydrothermal temperatures for 12 h with TEA (3 mL) and without NaOAC.

**Figure 8:**
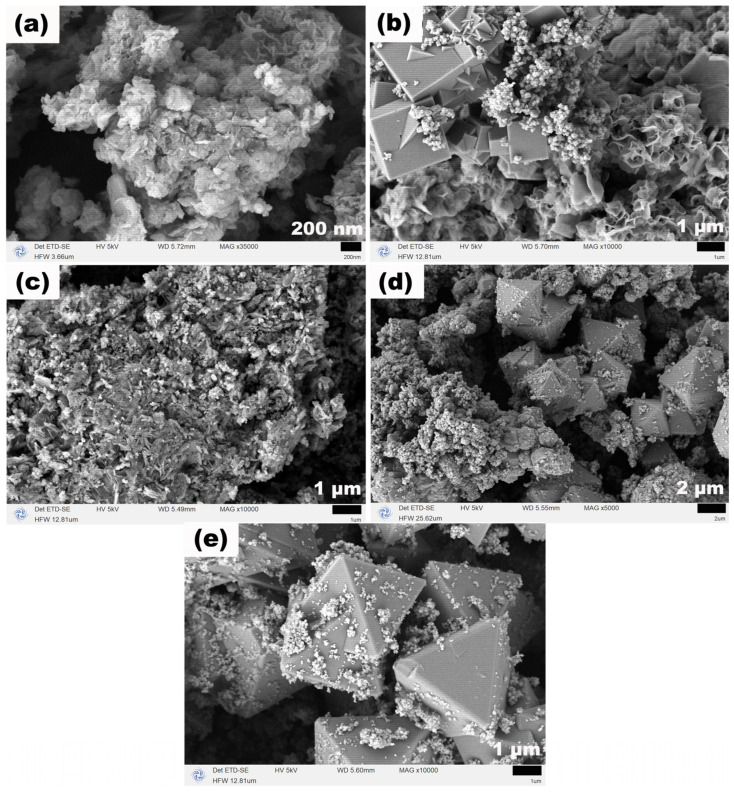
SEM images of samples synthesized at (**a**) 120, (**b**) 140, (**c**) 160, (**d**) 180 and (**e**) 200 °C for 12 h with TEA (3 mL) and without NaOAC.

Figure 7 and Figure 8 present the XRD patterns and SEM images of the samples synthesized with TEA (3 mL) alone, in the absence of NaOAC. Remarkably, the XRD patterns in Figure 7 show that all diffraction peaks of the samples corresponded well to the inverse spinel Fe_3_O_4_ phase (JCPDS No. 19-0629), with no detectable peaks corresponding to other phases such as FeOOH or Fe_2_O_3_. This indicates that pure-phase Fe_3_O_4_ was obtained across the entire hydrothermal temperature range (120–200 °C) with TEA alone, without the addition of NaOAC. The diffraction peak intensity was extremely low for the sample obtained at 120 °C, slightly increased at 140 °C, and became clearly discernible at 160 °C. The peak intensity increased markedly again at 180 °C and remained essentially unchanged at 200 °C.

Figure 8 presents the SEM images of the corresponding Fe_3_O_4_ samples, revealing an intriguing morphological evolution. At 120 °C, the product exhibited a flower-like morphology formed by the continuous growth and interweaving of irregular sheet-like structures with a thickness of approximately 20 nm. When the temperature increased to 140 °C, cubic structures with edge lengths of 0.5–2.5 μm appeared, coexisting with the flower-like morphology. At 160 °C, both the flower-like and cubic structures completely disappeared, replaced by micron-sized aggregates approximately 10 μm in size, which were formed by the aggregation of numerous irregular nanoparticles. At 180 °C, regular octahedral particles with edge lengths of about 2 μm emerged in the product, while particle aggregates were still present. When the temperature was further raised to 200 °C, the octahedral particles became more uniform and slightly larger, with edge lengths of approximately 3.8 μm; however, a small number of nanoparticles around 8 nm in size remained attached to the surfaces and surroundings of these octahedra. This series of remarkable morphological transformations demonstrates that TEA plays a crucial role in the crystal growth and morphological regulation of Fe_3_O_4_. As a strong complexing agent, TEA can form stable complexes with Fe^2+^ ions, thereby controlling the release rate of Fe^2+^ and subsequently influencing the nucleation and growth processes of the crystals. Even in the absence of the additional alkaline environment provided by NaOAC, TEA alone was able to guide the morphological evolution of Fe_3_O_4_ from flower-like structures at low temperatures, through cubic structures and aggregates, and finally to well-defined octahedra at high temperatures, underscoring its dominant role in morphology evolution.

#### 2.2.3. Time-Dependent Morphology Evolution with TEA Alone at 120 °C

**Figure 9:**
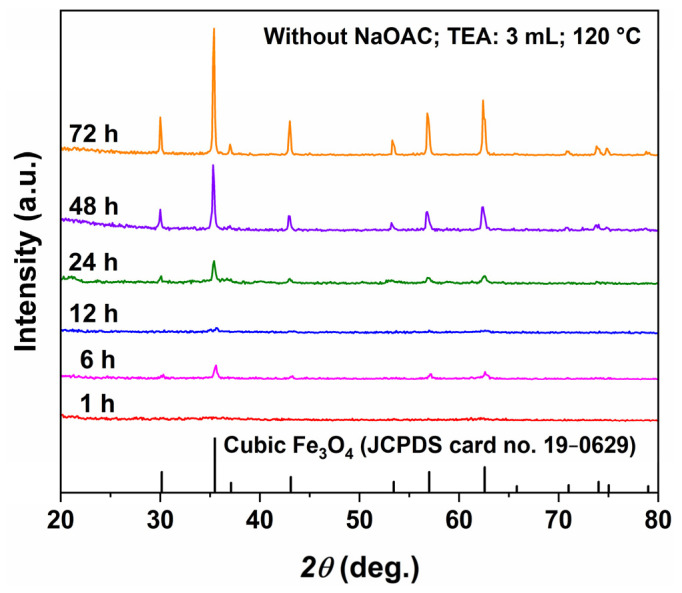
XRD patterns of samples synthesized at 120 °C for different hydrothermal time (1–72 h) with TEA (3 mL) and without NaOAC.

**Figure 10:**
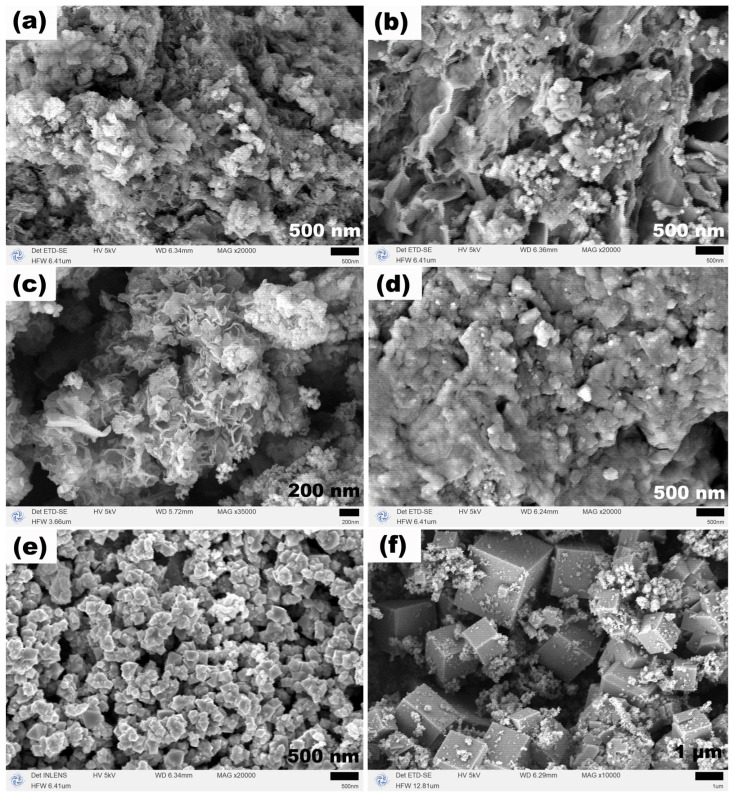
SEM images of samples synthesized at 120 °C for different hydrothermal time with TEA (3 mL) and without NaOAC: (1–72 h): (**a**) 1 h, (**b**) 6 h, (**c**) 12 h, (**d**) 24 h, (**e**) 48 h, and (**f**) 72 h.

To reveal the formation and evolution of the flower-like morphology, hydrothermal reactions were carried out at 120 °C for different durations (1, 6, 12, 24, 48, and 72 h) with a fixed TEA amount of 3 mL and without NaOAC. The corresponding XRD patterns and SEM images are shown in Figure 9 and Figure 10, respectively. The XRD results in Figure 9 show almost no diffraction peaks at 1 h, indicating that the product is mainly amorphous. At 6 h, broad diffraction peaks corresponding to inverse spinel Fe_3_O_4_ (JCPDS No. 19-0629) appear, suggesting low crystallinity. At 12 h, the peak intensity slightly decreases, possibly due to partial dissolution of the as-formed Fe_3_O_4_. After 24 h, the diffraction peaks gradually become sharper and more intense, indicating continuous improvement in crystallinity, and no other iron oxide phases are detected. This evolution supports the “dissolution–recrystallization” mechanism.

The SEM images in Figure 10 illustrate the morphological evolution: at 1 h, loose cotton-like aggregates are observed; at 6 h, a nascent flower-like structure assembled from nanosheets appears; at 12 h, the flower-like structure becomes more developed; at 24 h, the flower-like structure begins to dissolve, with small particles and the outlines of polyhedra appearing on the surface; at 48 h, the flower-like structure essentially disappears and is replaced by aggregates (ca. 300 nm) composed of nanoscale polyhedra (ca. 100 nm); at 72 h, a large number of micrometer-sized cubic particles (edge length up to ca. 3 μm) are formed, with a few nanoparticles still attached around them. This series of morphological transformations indicates that, with TEA alone, prolonged reaction time leads to the dissolution of the flower-like structure and its recrystallization into more stable cubic particles, further confirming the dissolution–recrystallization growth mechanism.

#### 2.2.4. Effect of TEA Dosage on Morphology with TEA Alone at 200 °C

**Figure 11:**
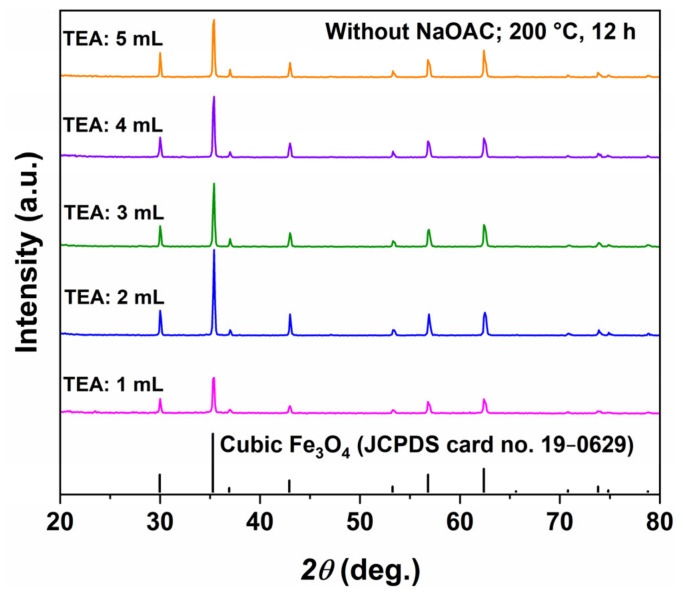
XRD patterns of Fe_3_O_4_ samples synthesized at 200 °C for 12 h with different TEA dosages (1–5 mL) and without NaOAC.

**Figure 12:**
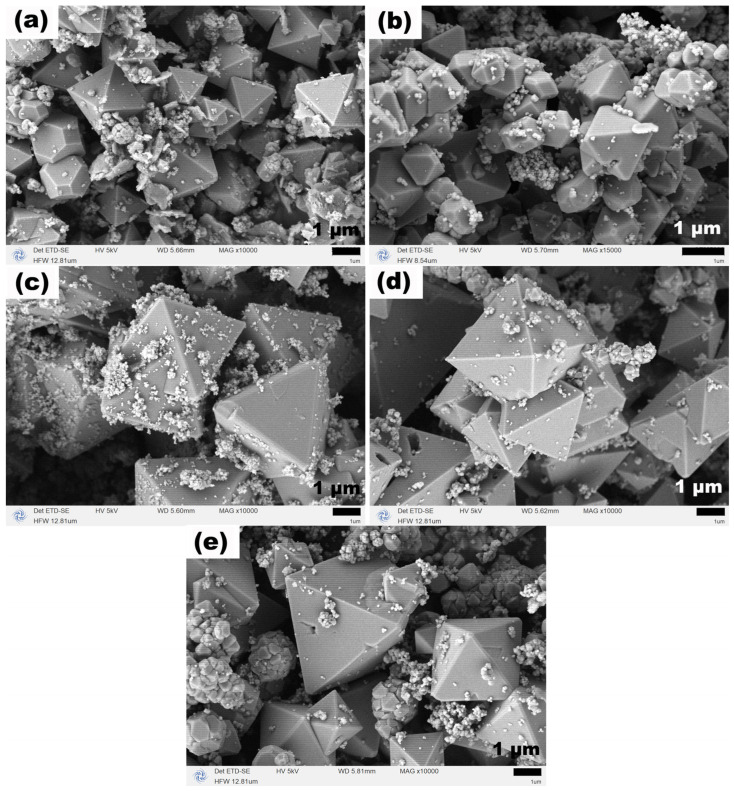
SEM images of Fe_3_O_4_ samples synthesized at 200 °C for 12 h with different TEA dosages: (**a**) 1 mL, (**b**) 2 mL, (**c**) 3 mL, (**d**) 4 mL, and (**e**) 5 mL.

To further investigate the morphology regulation ability of TEA alone, hydrothermal reactions were carried out at 200 °C for 12 h without NaOAC, while varying the TEA dosage (1, 2, 3, 4, and 5 mL). The corresponding XRD patterns and SEM images are shown in Figure 11 and Figure 12, respectively.

The XRD patterns in Figure 11 reveal that, for all TEA dosages, all diffraction peaks are consistent with inverse spinel Fe_3_O_4_ (JCPDS No. 19-0629) and no impurity peaks are detected, indicating that pure Fe_3_O_4_ can be obtained at 200 °C with as little as 1 mL of TEA. When the TEA dosage increases from 1 to 2 mL, the diffraction peaks become significantly sharper and more intense, with narrower full width at half maximum (FWHM), indicating improved crystallinity. Further increasing the TEA dosage to 3–5 mL leads to a slight decrease in peak intensity, but the peaks remain relatively sharp, suggesting that excess TEA may slightly inhibit the crystallization process.

The SEM images in Figure 12 show that at a TEA dosage of 1 mL, the product consists mainly of irregular polyhedral particles with unclear edges. At 2 mL, regular octahedral particles begin to appear, with some octahedra exhibiting flat tips, which may be an intermediate state transitioning from polyhedra to regular octahedra. At 3 and 4 mL, the product is almost entirely composed of regular octahedra with sharp edges and uniform size, with few attached nanoparticles. At 5 mL, regular octahedra still dominate, but a small number of nanoscale debris appear on the surfaces and surroundings. These results demonstrate that, in the absence of NaOAC at 200 °C, a TEA dosage in the range of 2–4 mL favors the formation of high-quality regular octahedral Fe_3_O_4_, while excessive TEA introduces a small amount of nanoscale debris.

### 2.3. Effect of TEA Dosage in the Presence of NaOAC (At 160 °C)

**Figure 13:**
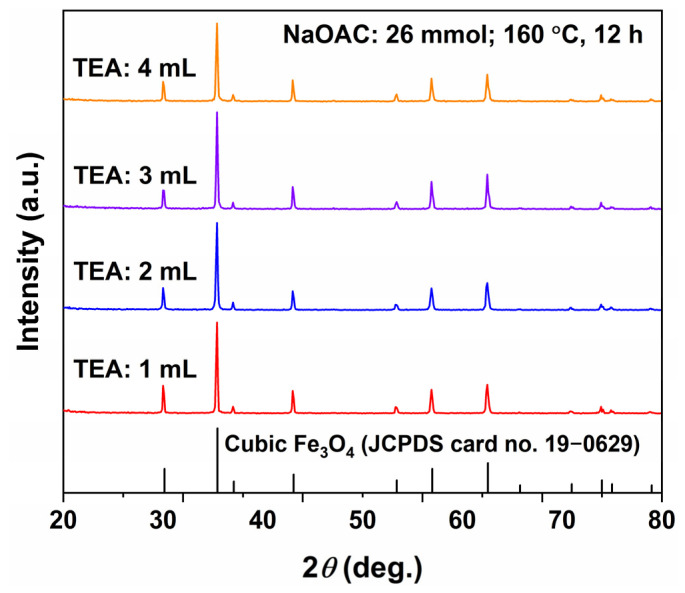
XRD patterns of Fe_3_O_4_ samples synthesized at 160 °C for 12 h with different TEA dosages (1–4 mL) and fixed NaOAC (26 mmol).

**Figure 14:**
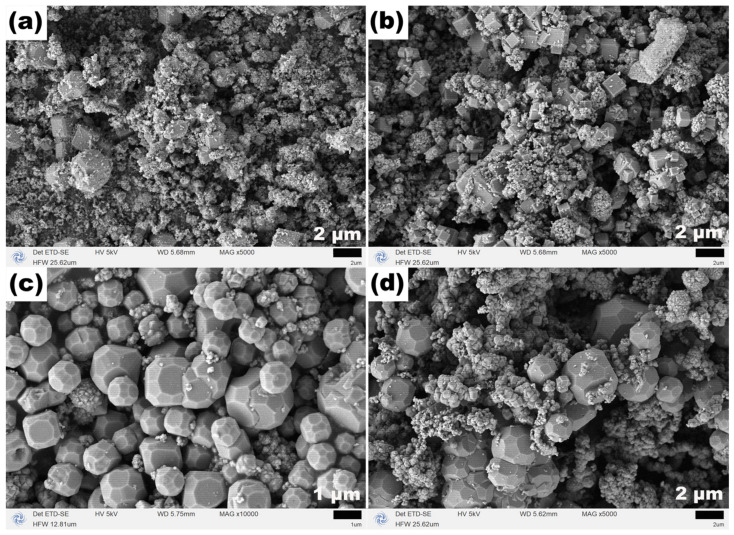
SEM images of Fe_3_O_4_ samples synthesized at 160 °C for 12 h with different TEA dosages: (**a**) 1 mL, (**b**) 2 mL, (**c**) 3 mL, (**d**) 4 mL, and fixed NaOAC (26 mmol).

To investigate the influence of TEA dosage on the crystallization process and morphological evolution of Fe_3_O_4_, a series of experiments were conducted with fixed NaOAC dosage (26 mmol) and hydrothermal conditions (160 °C, 12 h), while varying the TEA dosage from 1 to 4 mL. The phase composition, microstructure, and morphological characteristics of the obtained products were examined, and the corresponding results are presented in Figure 13 and Figure 14.

As shown in the XRD patterns in Figure 13, all diffraction peaks of the samples corresponded well to the inverse spinel Fe_3_O_4_ phase (JCPDS No. 19-0629), with no detectable peaks corresponding to other phases such as FeOOH or Fe_2_O_3_. This indicates that pure-phase Fe_3_O_4_ was obtained across the entire TEA dosage range of 1–4 mL. Notably, all samples exhibited well-defined and sharp diffraction peaks. The peak intensities of the samples obtained with 1–3 mL TEA were relatively similar, while a slight decrease in peak intensity was observed for the sample obtained with 4 mL TEA, suggesting that an excessive amount of TEA might have a certain influence on the crystallization process of Fe_3_O_4_.

Figure 14 presents the SEM images of the corresponding samples, revealing the significant influence of TEA dosage on the morphology of Fe_3_O_4_. When the TEA dosage was 1 mL, the product exhibited a mixed morphology consisting of various micron-sized polyhedral particles (less than 3 μm in size) and nanoparticles without specific morphology. As the TEA dosage increased to 2 mL, the amorphous nanoparticles significantly decreased, and cubic particles became the dominant morphology. At a TEA dosage of 3 mL, the product was predominantly composed of well-defined polyhedral structures, although a small number of aggregates assembled from nano-polyhedra could still be observed. When the TEA dosage was further increased to 4 mL, the number of nano-polyhedral aggregates increased markedly, becoming comparable to that of regular micron-sized polyhedra, and the morphology again tended toward a mixed state. This morphological evolution provides direct evidence for the role of TEA as a morphology-directing agent: an appropriate amount of TEA (3 mL) can regulate the crystal facet growth rate through complexation with Fe^2+^, promoting the preferential exposure of specific crystal facets and thereby inducing the formation of octahedral or cubic morphologies. In contrast, excessive TEA (4 mL) might lead to overly strong complexation, inhibiting sustained crystal growth and resulting in a large number of nano-polyhedra that fail to further assemble into micron-sized single crystals, instead existing as aggregates.

Based on the combined results of XRD analysis, quantitative calculations, and SEM observations, it can be concluded that TEA dosage plays a crucial regulatory role in the crystallization behavior and morphological evolution of Fe_3_O_4_. An appropriate amount of TEA (3 mL) favors the formation of micron-sized polyhedral Fe_3_O_4_ with relatively high crystallinity and well-defined morphology. However, excessive TEA (4 mL) perturbs the dissolution–recrystallization equilibrium, leading to decreased crystallinity, increased microstrain, and the formation of nano-polyhedral aggregates. This finding is consistent with the TEA-guided morphological evolution mechanism observed under NaOAC-free conditions, further confirming the dual function of TEA as both a complexing agent and a morphology-directing agent.

### 2.4. Effect of Reduced NaOAC Dosage

**Figure 15:**
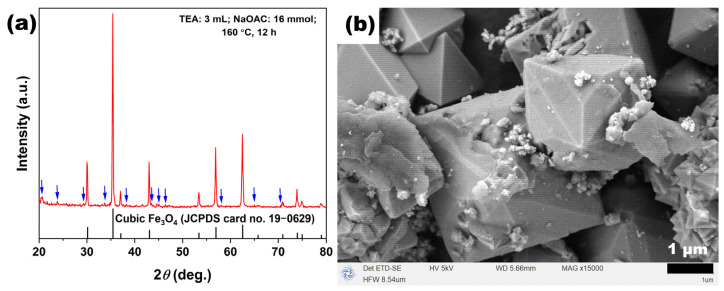
(**a**) XRD pattern and (**b**) SEM image of sample synthesized at 160 °C for 12 h with reduced NaOAC dosage (16 mmol) and fixed TEA (3 mL).

To further verify the role of NaOAC in the formation of Fe_3_O_4_, the NaOAC dosage was reduced from the standard condition (26 mmol) to 16 mmol while keeping other parameters constant (TEA 3 mL, 160 °C, 12 h). The phase composition and morphological characteristics of the obtained product are shown in Figure 15. The XRD pattern in Figure 15a reveals that, in addition to the characteristic diffraction peaks of inverse spinel Fe_3_O_4_ (JCPDS No. 19-0629), weak additional diffraction peaks were observed at positions indicated by the blue arrows. These peaks matched well with the characteristic peaks of hematite (*α*-Fe_2_O_3_, JCPDS No. 33-0664), indicating that when the NaOAC dosage was insufficient, the system failed to produce pure-phase Fe_3_O_4_, and a small amount of *α*-Fe_2_O_3_ impurity phase was generated in the product. The SEM image in Figure 15b shows that the product mainly consisted of micron-sized octahedral particles and aggregates assembled from nano-sized octahedra. Notably, a layer of attachment could be observed on the surfaces of some micron-sized octahedra, which, in combination with the XRD results, could be inferred as *α*-Fe_2_O_3_. This phenomenon suggests that the reduced NaOAC dosage led to insufficient alkalinity in the system, which was inadequate to completely suppress the oxidation of Fe^2+^ and the formation of Fe_2_O_3_, nor could it provide a suitable alkaline environment for the sufficient growth of Fe_3_O_4_ crystals.

**Figure 16:**
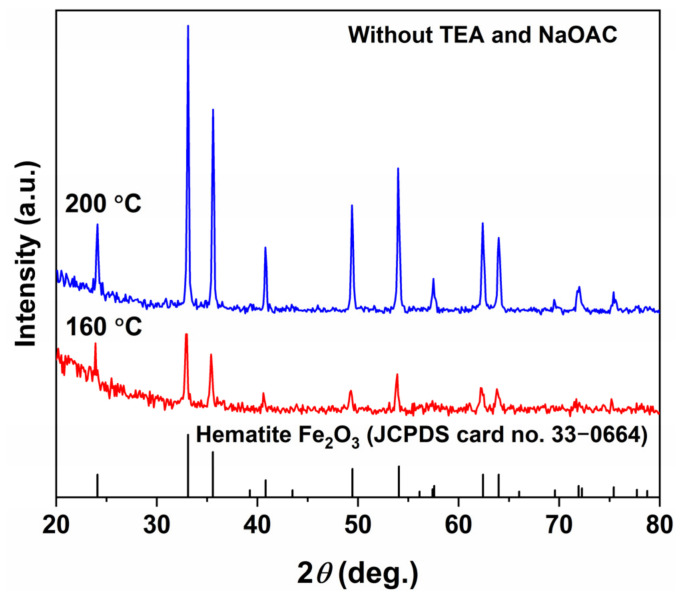
XRD patterns of control samples synthesized at 160 °C and 200 °C for 12 h in the absence of both TEA and NaOAC.

**Figure 17:**
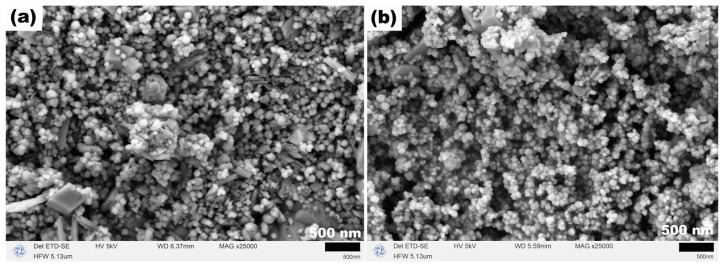
SEM images of control samples synthesized at (**a**) 160 °C and (**b**) 200 °C for 12 h in the absence of both TEA and NaOAC.

To further elucidate the synergistic roles of TEA and NaOAC in the formation of Fe_3_O_4_, control experiments were conducted in the complete absence of any additives at hydrothermal temperatures of 160 and 200 °C for 12 h (the 200 °C condition was included as a supplementary experiment to investigate the effect of temperature on the additive-free system). The XRD and SEM results of the obtained products are presented in Figure 16 and Figure 17. As shown in the XRD patterns in Figure 16, the products obtained at both 160 and 200 °C were single-phase *α*-Fe_2_O_3_ (JCPDS No. 33-0664), with the diffraction peak intensity of the product obtained at 200 °C being significantly higher than that obtained at 160 °C, indicating that elevated temperature favored the crystal growth of *α*-Fe_2_O_3_. The SEM images in Figure 17 reveal that the *α*-Fe_2_O_3_ products obtained at both temperatures mainly consisted of nanoscale spherical-like particles approximately 12 nm in size, interspersed with a small number of polyhedral particles, exhibiting a uniform but uncontrollable morphology.

### 2.5. Magnetic Property Analysis

**Figure 18:**
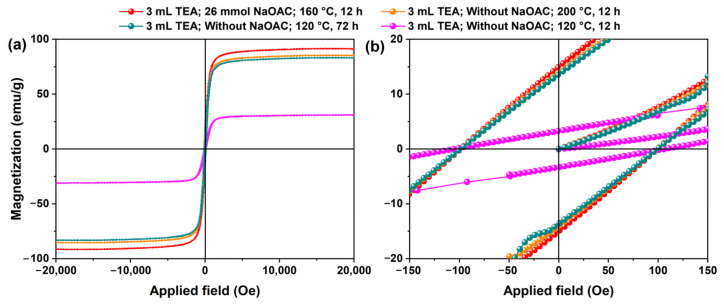
(**a**) Room-temperature magnetic hysteresis loops and (**b**) magnified view of the low-field region of Fe_3_O_4_ sample synthesized with different morphologies synthesized under various conditions: polyhedral (160 °C, 12 h, TEA 3 mL, NaOAC 26 mmol); regular octahedral (200 °C, 12 h, TEA 3 mL without NaOAC); cubic (120 °C, 72 h, TEA 3 mL without NaOAC); and flower-like (120 °C, 12 h, TEA 3 mL without NaOAC).

To systematically investigate the magnetic properties of Fe_3_O_4_ with different morphologies, room-temperature magnetic hysteresis loops were measured for four typical samples, and the results are shown in Figure 18. The four samples are: polyhedral Fe_3_O_4_ synthesized at 160 °C with TEA (3 mL) and NaOAC (26 mmol) (morphology shown in Figure 3c and Figure 14c); regular octahedral Fe_3_O_4_ synthesized at 200 °C with TEA (3 mL) and without NaOAC (Figure 8e); cubic Fe_3_O_4_ obtained at 120 °C with TEA (3 mL) and without NaOAC after 72 h (Figure 10f); and flower-like Fe_3_O_4_ obtained at 120 °C with TEA (3 mL) and without NaOAC after 12 h (Figure 8a and Figure 10c).

As shown in Figure 18a, all samples exhibit typical ferromagnetic behavior, but their saturation magnetization (Ms) values differ significantly. The polyhedral sample shows the highest Ms of 91.4 emu/g, very close to the theoretical value of bulk Fe_3_O_4_ (92 emu/g [27]), owing to its micrometer size and high crystalline perfection. The saturation magnetization of our polyhedral sample (91.4 emu/g) compares favorably with values reported for other Fe_3_O_4_ materials in the literature. Previous studies have reported Ms values ranging from approximately 60 to 80 emu/g for Fe_3_O_4_ microspheres, nanoparticles, and hollow structures synthesized by hydrothermal or related methods [28,29,30]. The slightly higher Ms observed in the present work may be attributed to the micrometer-sized single-crystal nature of our polyhedral particles, which helps to minimize surface spin disorder and magnetic dead layer effects that commonly reduce magnetization in nanocrystalline or polycrystalline samples. It should be noted, however, that differences in particle size, morphology, crystallinity, and measurement conditions across studies may also contribute to variations in the reported saturation magnetization values.

The regular octahedral and cubic samples give Ms values of 85.3 and 83.0 emu/g, respectively, slightly lower than that of the polyhedral sample, which may be attributed to a small number of attached nanoparticles and residual strain. The flower-like sample exhibits a much lower Ms of only 31.0 emu/g. This drastic reduction can be explained by two factors: first, the flower-like structure is assembled from nanosheets, introducing numerous grain boundaries and surface defects that enhance surface spin disorder and magnetic dead layer effects; second, its XRD diffraction peaks are significantly broader and weaker, indicating lower crystallinity, which limits magnetic ordering. Figure 18b presents a magnified view of the low-field region, revealing the coercivity (Hc) of the samples. All four samples exhibit coercivities of approximately 100 Oe, demonstrating typical soft magnetic characteristics [31]. Although a coercivity of about 100 Oe is not suitable for in vivo biomedical imaging or drug delivery that require superparamagnetic behavior, it is fully acceptable for applications such as magnetic separation, magnetic recording media, magnetic actuators, and certain catalytic recovery processes [32]. Among them, the polyhedral Fe_3_O_4_ combines high saturation magnetization with moderate coercivity, making it promising for the above non-biomedical magnetic applications [33].

## 3. Materials and Methods

### 3.1. Starting Materials

Ferrous sulfate heptahydrate (FeSO_4_∙7H_2_O, 99.95%), triethanolamine (TEA, 99.0%) and sodium acetate (NaOAC, 99.995%) were supplied by Aladdin Co., Ltd. (Shanghai, China); Ethanol (99.7%) was obtained from Chengdu Kelong Chemical Co., Ltd. (Chengdu, China); All reagents were utilized in their pristine form, without any additional processing, and purified water was employed throughout all experimental procedures.

### 3.2. Synthesis

A simple one-step hydrothermal process without any subsequent high-temperature roasting step was employed to prepare all samples. The detailed synthesis parameters, including additive combinations, hydrothermal temperatures, and reaction time, are summarized in Table 1. After the hydrothermal reaction, the same washing and separation procedure was applied to all samples. Briefly, the autoclave was allowed to cool to room temperature. Magnetic samples (e.g., Fe_3_O_4_) were collected by magnetic separation, while non-magnetic samples (e.g., *α*-Fe_2_O_3_) were collected by centrifugation (8000 rpm, 10 min). The collected precipitates were washed alternately with purified water and anhydrous ethanol three times (water first, then ethanol). In each washing step, the precipitates were dispersed in the washing solution under ultrasonication for 5 min, followed by separation according to the magnetic nature of the sample. Finally, the washed products were dried under vacuum at 60 °C for 24 h.

#### 3.2.1. Synthesis in the Presence of Both TEA and NaOAC (Temperature-Dependent Experiments)

2 mmol of FeSO_4_∙7H_2_O was dissolved in 15 mL of purified water. Separately, 3 mL of TEA was dissolved in 10 mL of purified water and added dropwise to the Fe^2+^ aqueous solution under continuous stirring for 5 min. Then, 26 mmol of NaOAC was added and stirred for another 5 min to obtain a green transparent solution. The solution was transferred into a 50 mL Teflon-lined stainless steel autoclave and heated at predetermined temperatures (120, 140, 160, 180, and 200 °C for 12 h.

#### 3.2.2. Synthesis in the Presence of NaOAC Only (Without TEA, Temperature-Dependent Experiments)

The same procedure as described in Section 3.2.1 was followed, except that no TEA was added.

#### 3.2.3. Synthesis in the Presence of TEA Only (Without NaOAC, Temperature-Dependent Experiments)

The same procedure as described in Section 3.2.1 was followed, except that no NaOAC was added.

#### 3.2.4. Synthesis with Different TEA Dosages in the Presence of NaOAC (At 160 °C)

The same procedure as described in Section 3.2.1 was followed, except that the TEA dosage was varied (1, 2, 3, and 4 mL) and the hydrothermal temperature was fixed at 160 °C.

#### 3.2.5. Synthesis with Reduced NaOAC Dosages (At 160 °C)

The same procedure as described in Section 3.2.1 was followed, except that the NaOAC dosage was reduced from 26 mmol to 16 mmol, and the hydrothermal temperature was fixed at 160 °C.

#### 3.2.6. Time-Dependent Synthesis with TEA Only (Without NaOAC, at 120 °C)

The same procedure as described in Section 3.2.1 was followed, except that no NaOAC was added, the TEA dosage was fixed at 3 mL, the hydrothermal temperature was fixed at 120 °C, and the reaction time was varied (1, 6, 12, 24, 48, and 72 h).

#### 3.2.7. TEA Dosage-Dependent Synthesis with TEA Only (Without NaOAC, at 200 °C)

The same procedure as described in Section 3.2.1 was followed, except that no NaOAC was added, the TEA dosage was varied (1, 2, 3, 4, and 5 mL), and the hydrothermal temperature was fixed at 200 °C for 12 h.

#### 3.2.8. Control Synthesis in the Absence of Both TEA and NaOAC (Temperature-Dependent Experiments)

The same procedure as described in Section 3.2.1 was followed, except that no TEA and no NaOAC were added. The hydrothermal temperatures were 160 and 200 °C for 12 h.

### 3.3. Characterization

The phase composition of the samples was analyzed using a DX-2700 X-Ray diffractometer (XRD; Dandong Haoyuan Instrument Co., Ltd., Dandong, China). The morphology of the samples was characterized by a SEM5000 Scanning Electron Microscope (SEM; CIQTEK Co., Ltd., Hefei, China). The microstructure and crystallographic structure of the samples were examined using a Tecnai G2 F30 transmission electron microscope (TEM; FEI, Hillsboro, OR, USA). High-resolution TEM (HRTEM) images and selected area electron diffraction (SAED) patterns were obtained to determine the lattice fringes and single-crystalline nature of the particles. The magnetic hysteresis loops were measured at room temperature using a SQUID–VSM magnetometer (Vibrating Sample Magnetometer; MPMS-3, Quantum Design, San Diego, CA, USA). The functional groups of the samples were examined by a Fourier transform infrared spectrometer (FTIR; FTIR-7600, Tianjin Gangdong Sci. and Tech. Co., Ltd., Tianjin, China). The surface composition and binding energies of the elements were determined by X-ray photoelectron spectroscopy (XPS; ESCALAB 250, Thermo Scientific, Waltham, MA, USA).

## 4. Conclusions

Using FeSO_4_·7H_2_O as a single iron source, morphology regulation of Fe_3_O_4_ with tunable morphologies was successfully achieved through a one-step hydrothermal method in a TEA–NaOAC synergistic system. While pure-phase Fe_3_O_4_ can be obtained using TEA alone, the introduction of NaOAC provides a mild alkaline environment that synergistically works with TEA to further promote anisotropic crystal growth and achieve a wider range of morphological control. In this synergistic system, TEA controls the release rate of Fe^2+^ and inhibits its oxidation via complexation, while NaOAC provides an alkaline environment that facilitates crystal growth. In the TEA-only system, by adjusting the temperature and reaction time, the morphology evolves from flower-like (120 °C, 12 h) to cubic (140 °C, 12 h) and finally to regular octahedral (200 °C, 12h). Time-dependent experiments (120 °C, 1–72 h) reveal the complete dissolution–recrystallization pathway from flower-like to cubic structures, where nanoscale polyhedral aggregates appear at 48 h and micrometer-sized cubic particles form at 72 h. TEA dosage-dependent experiments (200 °C, 1–5 mL) show that 2–4 mL of TEA favors the formation of high-quality regular octahedra, while excess TEA (5 mL) introduces nanoscale debris. In the TEA-NaOAC system, well-defined polyhedral Fe_3_O_4_ is obtained at 160 °C, and TEM together with SAED confirms that these polyhedral particles are micrometer-sized single crystals. Magnetic measurements give saturation magnetization values of 91.4, 85.3, 83.0, and 31.0 emu/g for the polyhedral, regular octahedral, cubic, and flower-like Fe_3_O_4_, respectively; the micrometer-sized single crystals (polyhedral) exhibit an Ms value closest to the bulk theoretical value (92 emu/g), and all samples show coercivities of approximately 100 Oe. The TEA–NaOAC synergistic system established in this study provides a systematic experimental basis and a regulatory strategy for the preparation of magnetic Fe_3_O_4_ with high saturation magnetization and moderate coercivity using a single iron source.

## Figures and Tables

**Table 1:** Experimental parameters for Fe_3_O_4_ synthesis with different additive combinations and hydrothermal temperatures.

Section	Experimental Parameters	Experimental Flow Chart
3.2.1	TEA (3 mL) + NaOAC (26 mmol); 120–200 °C, 12h	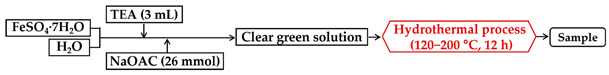
3.2.2	NaOAC (26 mmol), no TEA; 120–200 °C, 12 h	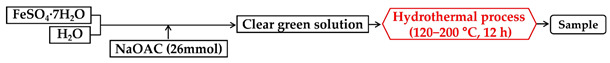
3.2.3	TEA (3 mL), no NaOAC; 120–200 °C, 12 h	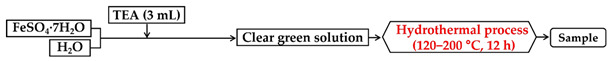
3.2.4	TEA (1–4 mL) + NaOAC (26 mmol); 160 °C, 12 h	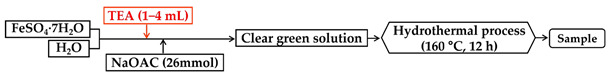
3.2.5	TEA (3 mL) + NaOAC (16 mmol); 160 °C, 12 h	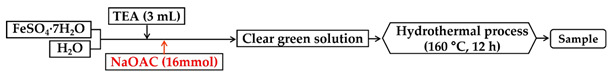
3.2.6	TEA (3 mL), no NaOAC, 120 °C, 1–72 h	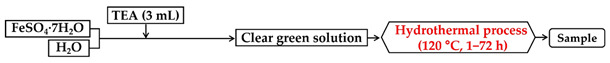
3.2.7	TEA (1–5 mL), no NaOAC, 200 °C, 12 h	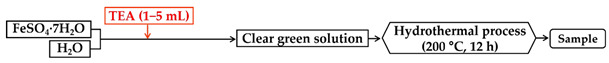
3.2.8	no TEA, no NaOAC; 160 and 200 °C, 12 h	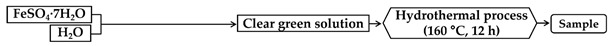

## Data Availability

The original contributions presented in this study are included in the article. Further inquiries can be directed to the corresponding authors.
